# Phenomics‐Based Discovery of Novel Orthosteric Choline Kinase Inhibitors

**DOI:** 10.1002/anie.202420149

**Published:** 2025-01-13

**Authors:** Ludwig G. Bauer, Jennifer A. Ward, Laura Díaz‐Sáez, Yvonne Sundström, Tuomas Tolvanen, Juan Carlos Alarcón Barrera, Sarantos Kostidis, Catherine M. Rogers, Ioanna Panagakou, Usha Singh, Elisabeth M. Rothweiler, Alejandro Gonzalez Orta, H. Ümit Kaniskan, Jianping Hu, Jian Jin, Sonja Sievers, Herbert Waldmann, Martin Giera, Michael Sundström, Louise Berg, Kilian V. M. Huber

**Affiliations:** ^1^ Centre for Medicines Discovery Nuffield Department of Medicine University of Oxford Oxford OX3 7FZ UK; ^2^ Target Discovery Institute Nuffield Department of Medicine University of Oxford Oxford OX3 7FZ UK; ^3^ Structural Genomics Consortium Karolinska Institutet 17176 Solna Sweden; ^4^ Department of Medicine Solna Karolinska Institutet and Karolinska University Hospital 17176 Stockholm Sweden; ^5^ Pelago Bioscience AB 17165 Solna Sweden; ^6^ Center for Proteomics and Metabolomics Leiden University Medical Center 2333ZA Leiden The Netherlands; ^7^ Mount Sinai Center for Therapeutics Discovery Departments of Pharmacological Sciences Oncological Sciences and Neuroscience Tisch Cancer Institute Icahn School of Medicine at Mount Sinai New York NY 10029 USA; ^8^ Compound Management and Screening Center at the Max Planck Institute of Molecular Physiology 44227 Dortmund Germany; ^9^ Department of Chemical Biology Max Planck Institute of Molecular Physiology 44227 Dortmund Germany

**Keywords:** cell painting, chemical probes, chemical proteomics, metabolomics, phenotypic

## Abstract

Choline kinase alpha (CHKA) is a central mediator of cell metabolism linked to cancer and immune regulation. Cellular and clinical evaluation of CHKA has been hampered by challenges in the development of drug‐like choline kinase inhibitors. Here, we identify CHKA as an unexpected off‐target of histone methyltransferase inhibitors using an integrated phenomic approach. We confirm CHKA as a direct protein target of the aminoquinazolines UNC0638 and UNC0737 using a combination of chemoproteomic, biochemical, cellular, and metabolic profiling assays, possibly explaining the previously reported discrepancies observed for different G9a/GLP inhibitor scaffolds in cellular assays. Using primary human cell model systems, we discover that CHKA modulation impairs IgG secretion and B‐cell maturation consistent with the notion that choline metabolism plays an important role in immune signalling. Co‐crystal structures of UNC0638 and UNC0737 with CHKA unravel an unexpected binding mode and suggest the inhibitors as attractive starting points for the development of selective chemical tools to further explore the biological role of CHKA in cancer and immune metabolism.

## Graphical abstract

Using an integrated phenomic approach we identified choline kinase alpha (CHKA) as an unexpected off‐target of the methyltransferase inhibitor UNC0638 and its negative control UNC0737, shedding light on their mechanism of action. This discovery offers a drug‐like starting for CHKA inhibitor development and investigating its role in immune metabolism.

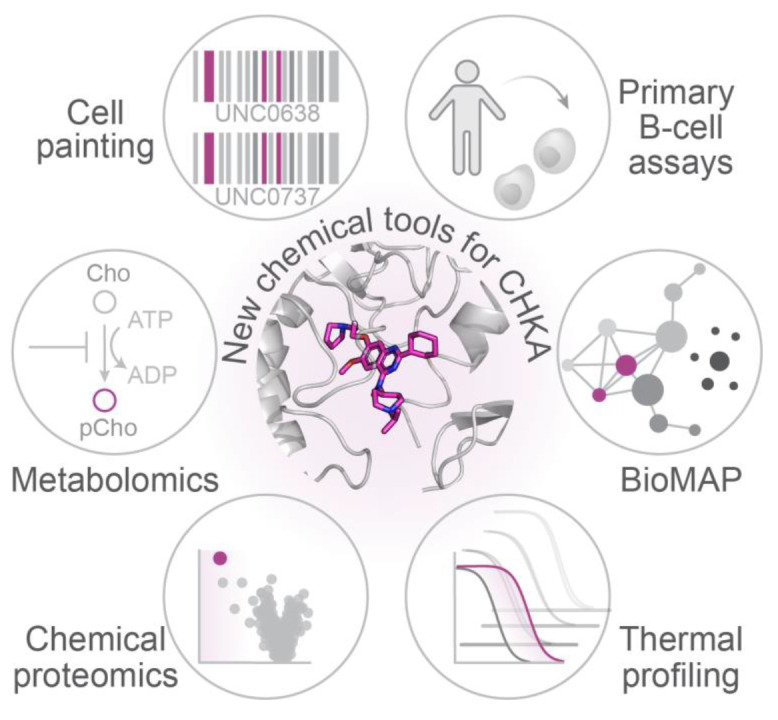

## Introduction

Chemical probes are versatile and powerful tools in biological research allowing for pre‐clinical assessment of potential drug targets and the discovery of new biology.[^1^, ^2^] High selectivity, broad annotation, availability of distinct chemotypes and companion negative control compounds are key to enable interpretation of cellular phenotypes. The paralogues EHMT1 (euchromatic histone lysine methyltransferase 1, also known as GLP) and EHMT2 (euchromatic histone lysine methyltransferase 2, also known as G9a) are two structurally related enzymes catalysing mono‐ and dimethylation of K9 on histone 3 (H3K9). Both proteins can also methylate a variety of other non‐histone substrates such as the tumour suppressor p53, sirtuin 1 (SIRT1), myogenic differentiation 1 (MyoD), chromodomain Y‐like protein (CDYL1) and widely‐interspaced zinc finger‐containing protein (WIZ).^[3]^ Dysregulation of G9a and/or GLP has been implicated in many human diseases such as cancer, inflammatory diseases, and neurodegenerative conditions. To date, several potent and selective small‐molecule inhibitors have been developed. BIX01294, UNC0638, UNC0642, and A‐366 have been screened extensively in both cellular and in vivo studies to gain insight into the biological functions of G9a and GLP.[^4^, ^5^, ^6^] However, UNC0638 has been found to exhibit strong antiproliferative effects against cancer cells, whereas other complementary G9a/GLP chemical probes such as A‐366 have failed to reproduce these phenotypes.^[7]^ These observations suggest additional unknown targets beyond G9a and GLP. Here, we use an integrated phenomic profiling approach to uncover choline kinase alpha (CHKA) as a potent and unexpected off‐target of UNC0638 and its negative control compound UNC0737. Unrelated to EHMT1/2, CHKA is a key enzyme in phosphatidylcholine metabolism and has been proposed as an anti‐cancer target, with one inhibitor previously evaluated in clinical trials.^[8]^ We confirm CHKA cellular target engagement by UNC0737 and UNC0638 resulting in perturbation of phosphocholine metabolism. Structural analysis by X‐ray crystallography revealed an unexpected binding mode via the choline binding site and suggests UNC0737 as a potent and selective tool compound to explore CHKA biology. Consistent with previous studies examining the role of CHKA in regulating immune metabolism,[^9^, ^10^] we observe CHKA‐dependent reduction of IgG secretion in primary immune cell assays. Collectively, our findings provide new avenues for the development of selective chemical tools to probe phosphocholine metabolism and explore the role of CHKA in both physiology and disease.

## Results and Discussion

**Figure 1:**
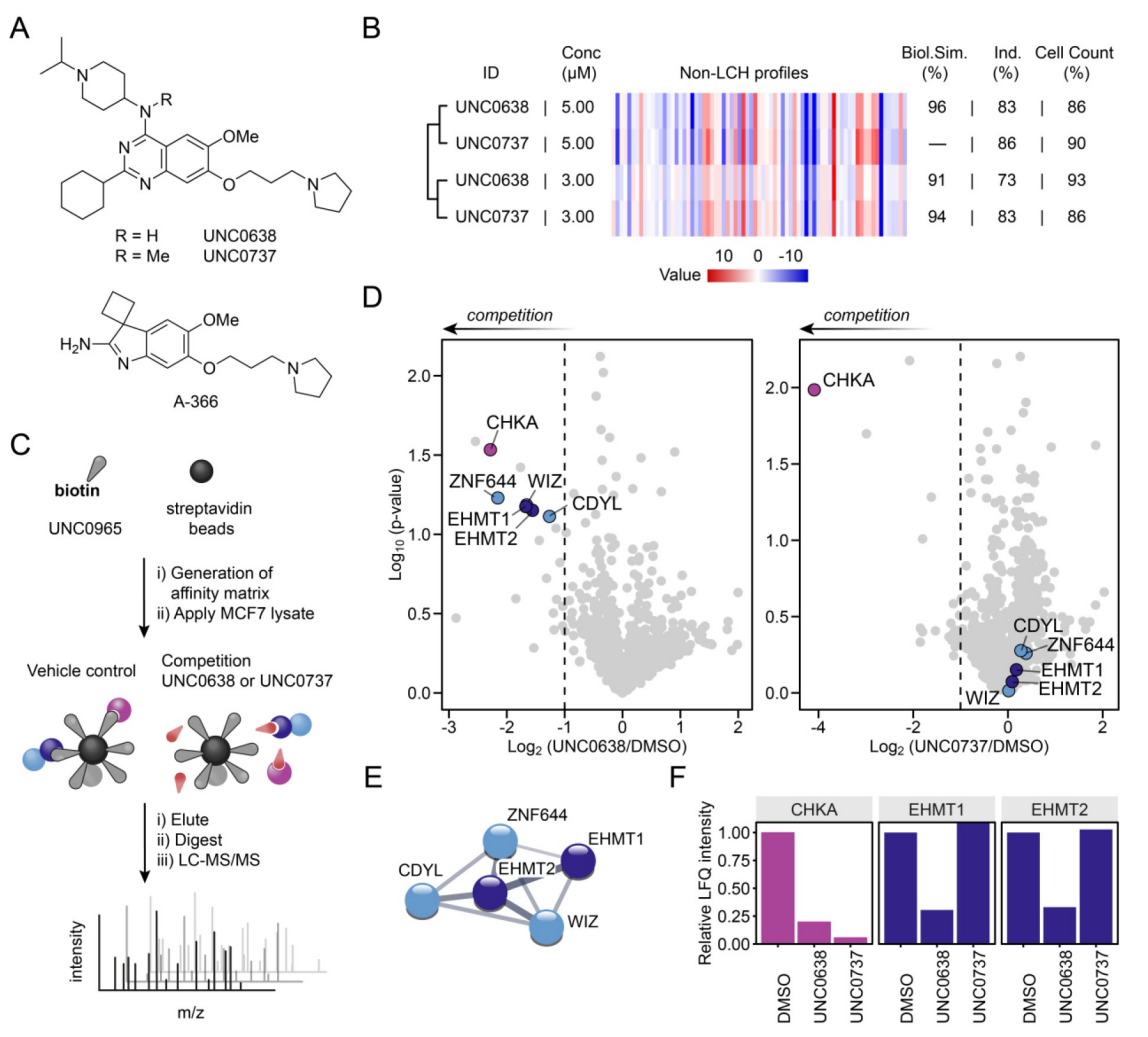
Identification of CHKA as an off‐target of UNC0638 and UNC0737 by phenotypic and chemoproteomic profiling. A) Chemical structures of UNC0638, UNC0737 and A‐366. B) Cell painting results showing high similarity for the methyltransferase‐active UNC0638 as well as the negative control UNC0737 in U2OS cells. Non‐LCH: Non‐lysosomotropic features. C) Affinity‐based chemoproteomics workflow. D) Competitive chemoproteomic data reveal CHKA as a shared off‐target for both UNC0638 and UNC0737 in MCF7 lysates. Dashed lines represent log_2_ competition<‐1 (p‐values from two‐sided two sample t‐test, *n*=2). 1,473 proteins were identified. E) Protein‐protein interaction network of EHMT1/2 complex partners (adapted from string‐db.org). F) Comparison of mean LFQ intensities. As expected, the G9a/GLP‐inactive UNC0737 does not compete with EHMT1/2 binding in contrast to the active compound UNC0638.

Following a previous report describing phenotypic discrepancies between chemically distinct G9a/GLP inhibitors,^[7]^ we performed 72 h cell viability assays in MCF7, MDA‐MB‐231, HEK293, HepG2, U2OS and K562 cells with the G9a/GLP chemical probe UNC0638 and its methyltransferase‐inactive companion negative control compound UNC0737 (Figure 1A). Surprisingly, we observed antiproliferative effects for both compounds whereas the structurally distinct A‐366 was less efficient in inducing cancer cell death (Figure 1A, Figure S1). To further investigate potential shared phenotypes between the active and inactive G9a/GLP probe more broadly, we conducted Cell Painting Assays (CPA) with UNC0638 and UNC0737 in U2OS cells.[^11^, ^12^] We compared morphological fingerprints of multiple parameters to assess overall biological similarity (Biol. Sim.) (Figure 1B). Interestingly, both UNC0638 and the negative control compound UNC0737 showed high induction (Ind.; % of parameters that exhibited significant changes) and clustered together with >90 % biosimilarity. These results strongly support the notion that the two compounds share common off‐target(s) that are driving the observed phenotypes across distinct cell lines.

To identify the molecular target(s) and elucidate the mechanism of action of UNC0638 and UNC0737, we performed affinity‐based chemoproteomics.[^13^, ^14^] We immobilised UNC0965, a biotinylated analogue of UNC0638 (Figure S2),^[15]^ on streptavidin‐coated sepharose beads to enrich binding proteins from whole MCF7 cell lysates. UNC0636 or UNC0737 were added as competitors in control experiments to identify specific binders (Figure 1C, D). In case of UNC0638, we observed strong enrichment and competition for its cognate targets EHMT1 (GLP) and EHMT2 (G9a). In addition, known complex partners such as WIZ,^[16]^ CDYL,^[17]^ and ZNF644^[18]^ were co‐purified (Figure 1E). Confirming previous biochemical data, competition with the negative control compound UNC0737 did not ablate binding of EHMT1 (GLP), EHMT2 (G9a) and related interactors to the affinity matrix (Figure 1D, F). Unexpectedly, we discovered choline kinase alpha (CHKA) as a shared off‐target of UNC0638 and UNC0737 (Figure 1D). Comparison of LFQ intensities indicated even stronger competition for CHKA by UNC0737 (Figure 1F). CHKA binding to the affinity matrix was validated by immunoblotting using a specific antibody (Figure S3).

**Figure 2:**
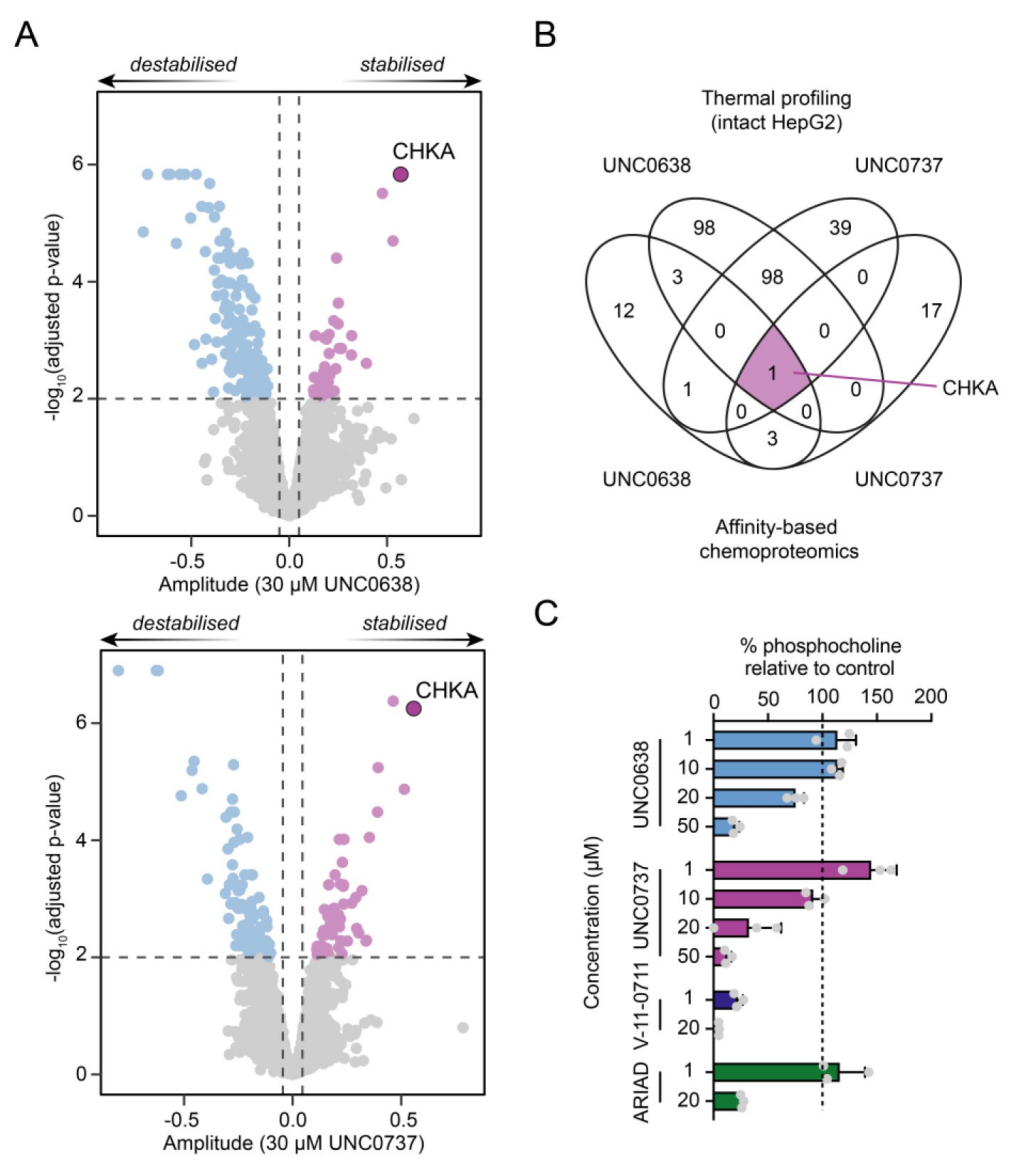
Thermal profiling by PISA and metabolomics confirm intracellular CHKA target engagement. A) UNC0638 and UNC0737 engage and stabilise CHKA in intact HepG2 cells. Dashed lines represent amplitude>0.05,<‐ 0.05 and log_10_(adjusted p‐values)>2 (two‐sided two sample t‐test, Benjamini‐Hochberg corrected, *n*=3). 5,635 proteins were identified. B) Comparison of significantly changed proteins between affinity‐based chemoproteomics (strongly competed proteins with FC<‐1) in MCF7 cells and thermal profiling (significantly changing proteins log_10_(p‐value)>2) of intact HepG2 cells. C) Effects of UNC0638 and UNC0737 on choline metabolism in MCF7 cells. NMR analysis of intracellular choline and phosphocholine (pCho) levels relative to vehicle control following incubation with indicated compounds. No change (100 %) is marked with a dashed line. Data are shown as mean±SD from three replicates (*n*=3).

To further corroborate our findings, we next performed the proteome integral solubility alteration (PISA) assay in HepG2 human hepatoma cells that express high levels of CHKA. Conceptually building on the cellular thermal shift assay (CETSA) with mass spectrometry based detection,[^19^, ^20^] PISA leverages compound‐mediated stabilisation of target proteins without the need of chemical modification in intact cells. Confirming our chemoproteomic results, we observed significant stabilisation of CHKA by both UNC0638 and UNC0737 in intact HepG2 cells (Figure 2A). Additional PISA experiments in K562 lysates suggested CHKA engagement independent of the cellular background (Figure S4).

CHKA catalyses the phosphorylation of choline and thereby plays a key role in phospholipid metabolism. We performed targeted NMR metabolite analysis of MCF7 cells treated with UNC0638 and UNC0737 evaluating phosphocholine (pCho), sn‐glycerophosphocholine (GPC), and total choline (Figure 2B, S5). Both compounds demonstrated a dose‐dependent reduction of pCho formation relative to control, similar to the previously published CHKA inhibitors V‐11‐0711^[21]^ and compound 67,^[6]^ hereafter called “ARIAD” (Figure 2B).

**Figure 3:**
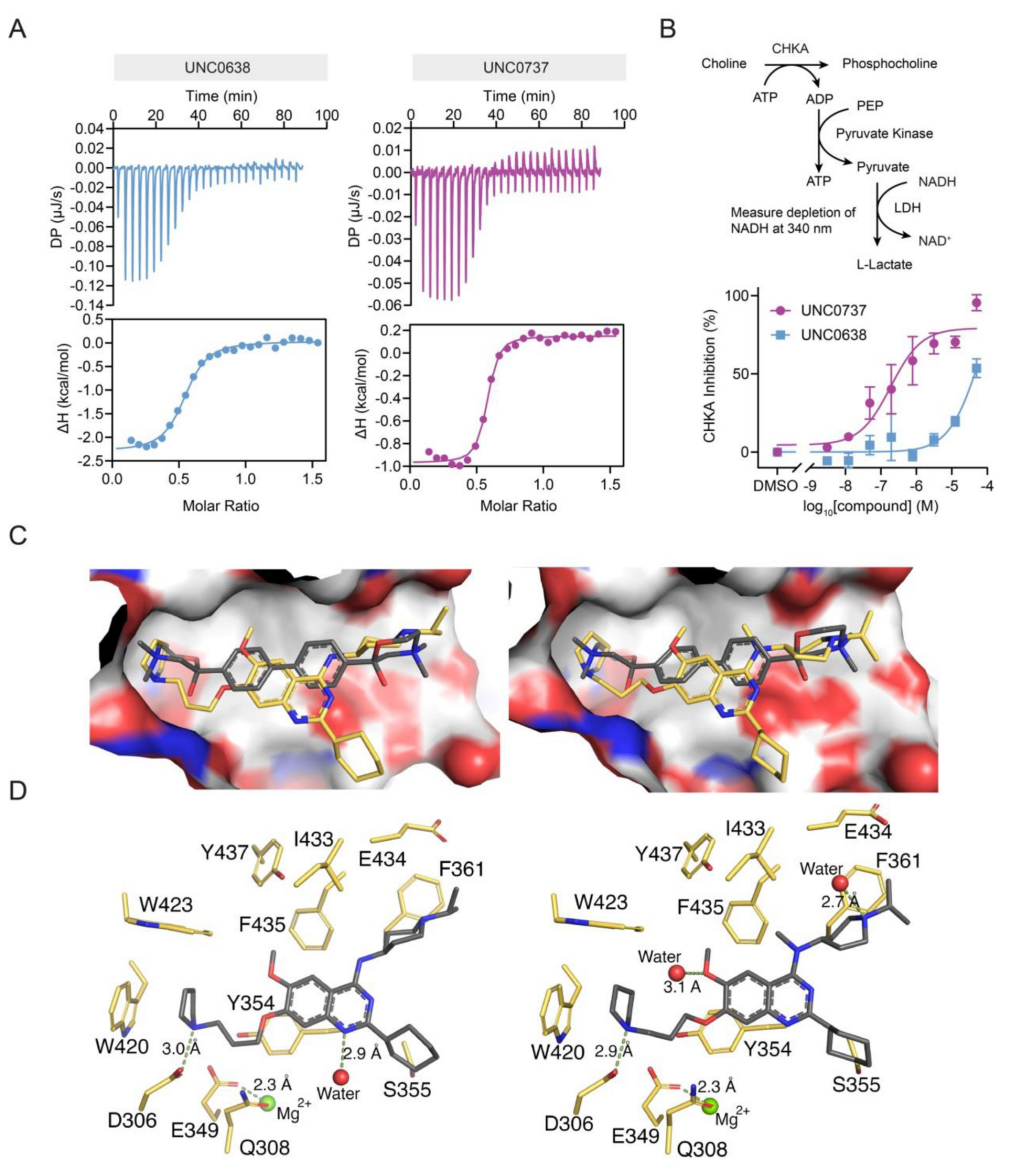
Confirmation of CHKA as a direct target of UNC0638 and UNC0737. A) ITC results for UNC0638 (*K*_D_=270±51 nM) and UNC0737 (*K*_D_=59.3±14 nM) using recombinant human CHKA. B) UNC0638 and UNC0737 inhibit CHKA enzymatic activity in a dose‐dependent manner. Data are shown as mean±SD of three replicates. Graph is representative of two independent biological replicates (*n*=2). C) Co‐crystal structures of UNC0638 (PDB: 8BI6) and UNC0737 (PDB: 8BI5) bound to CHKA. UNC0737 and UNC0638 (coloured in yellow) both occupy the HC3 binding site (PDB: 3F2R). HC3 is coloured in dark grey. D) Stick representation with residues contributing to compound binding.

Next, we sought to further characterise the molecular underpinnings of the interaction between UNC0737, UNC0638, and CHKA. To confirm direct binding, we expressed and purified recombinant human CHKA and performed isothermal calorimetry (ITC). Remarkably, we determined a *K*_D_ value of 270 nM for UNC0638 validating CHKA as a high affinity target (Figure 3A). In line with our chemoproteomics results, UNC0737 was even more potent with a *K*_D_ value of 59.3 nM, comparable to the previously reported CHKA inhibitor hemicholinium‐3 (HC3) (*K*_D_=40.6 nM, Figure S6A, B). Further analysis by differential scanning fluorimetry (DSF) demonstrated thermal stabilisation of CHKA in the presence of UNC0638 and UNC0737, but not for A‐366 (Figure S6C,D). We also profiled additional inhibitors, sharing the amino‐quinazoline scaffold, like BIX‐01294 and UNC0642, which also exhibited CHKA stabilisation albeit to lesser degree than UNC0737, thus providing further insights into CHKA SAR (Figure S6D,E). To see if direct binding of UNC0638 and UNC0737 to CHKA would translate into inhibition of enzymatic activity, we next conducted PK/LDH coupled choline kinase enzyme assays monitoring substrate turnover.^[22]^ Indeed, both UNC0638 and UNC0737 suppressed CHKA catalytic activity in a concentration‐dependent manner in line with the metabolomics results (Figure 3B).

In order to elucidate the molecular interaction of UNC0638 and UNC0737 with CHKA, we co‐crystallised CHKA with both compounds. The structures revealed that UNC0638 (PDB: 8BI6) and UNC0737 (PDB: 8BI5) bind to the choline binding site of CHKA (Figure 3C, Table S1). Both structures presented two protein molecules in the asymmetric unit with one showing higher B‐factors at the ligand binding sites indicating higher flexibility in that region. This wider and more flexible pocket allows the compounds to bind with two distinct conformations. In case of UNC0638 and UNC0737, important interactions are observed with Y354, W423, F261, and W420. UNC0737 is able to exploit two additional interactions with water molecules which may explain the higher potency observed in ITC and thermal stabilisation experiments (Figure 3D).

**Figure 4:**
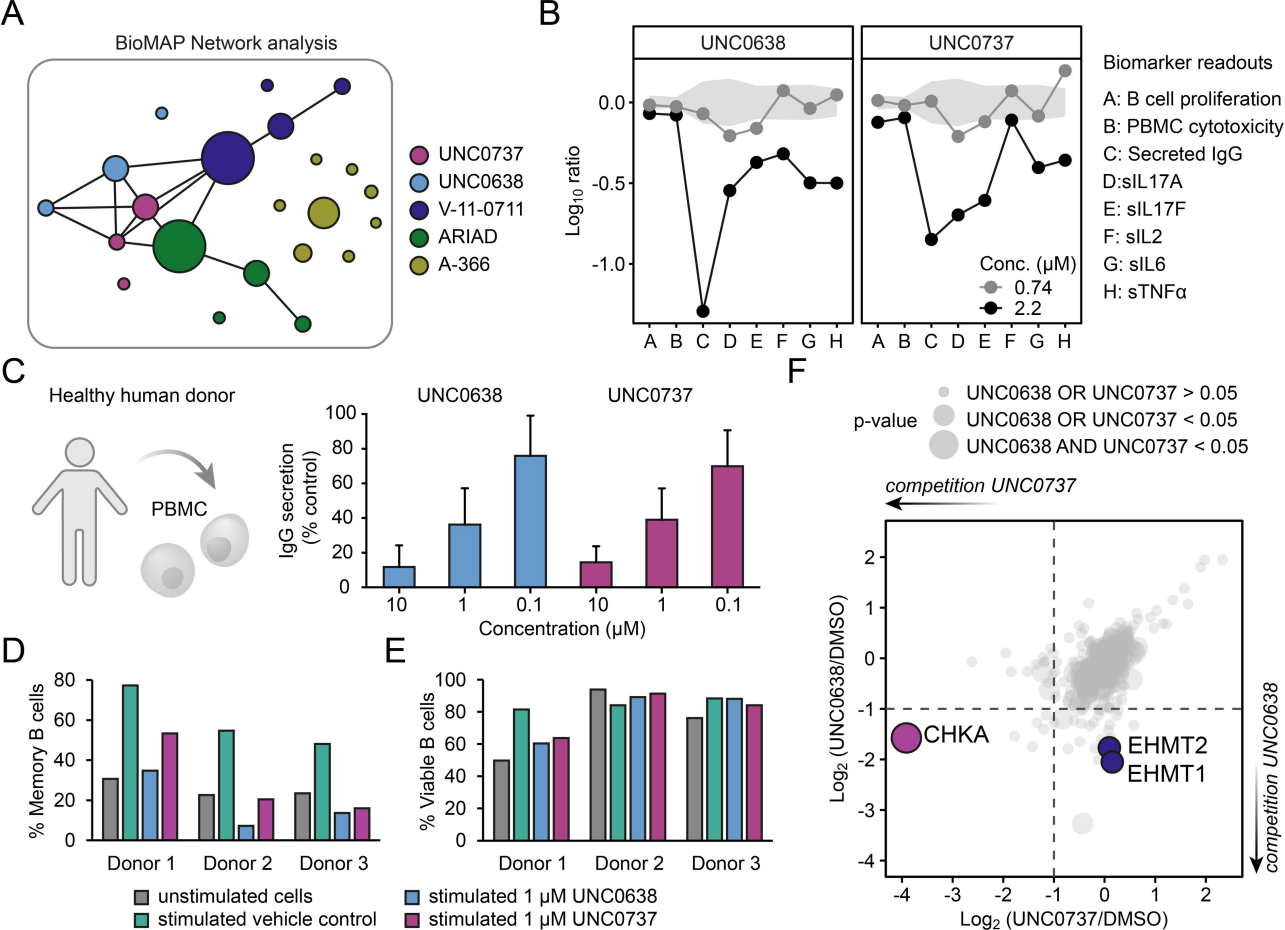
Effect of CHKA inhibitors on immune signalling. A) Network analysis of BioMAP profiling using indicated inhibitors over all 12 tested systems. CHKA inhibitors cluster together over multiple concentrations indicating mechanistic similarity in contrast to the CHKA‐inactive A‐366 (internal BioMAP control). Line indicates Pearson's correlation of full BioMAP profiles >0.7. Circle size represents concentration of compounds. B) BioMAP B cell profiling results indicating a common response via reduction of secreted IgG and sIL‐17A. The Y‐axis represents a log‐transformed ratio of the biomarker readouts for the drug‐treated sample (*n*=1) over vehicle controls (n ≥6). The grey region around the y‐axis represents the 95 % significance envelope generated from BioMAP historical vehicle controls. C) UNC0638 and UNC0737 reduce stimulated PBMC secretion of IgG, expressed as % of vehicle control in cell cultures from healthy blood donors. Data are shown as mean±SD calculated from seven donors (*n*=7). D) UNC0638 and UNC0737 reduce stimulation‐induced B cell maturation. The percent of CD27^+^IgD^−^ memory B cells of live CD19^+^ B cells is depicted for three different healthy donors, analysed after 6 days of culture in absence or presence of stimulation. E) Cell viability results for UNC0638 and UNC0737. The percent of live CD19^+^ B cells is depicted for three different healthy donors, analysed after 6 days of culture in absence or presence of stimulation. F) Correlation of competitive chemoproteomic data of UNC0638 or UNC0737 in PBMC lysates using UNC0965 affinity beads.

Having confirmed that UNC0638 and UNC0737 are potent *bona fide* CHKA inhibitors, we next turned our attention to further investigate the role of CHKA in cellular signalling. While CHKA has been studied extensively in the context of malignant disease, there has been a growing acknowledgment of its relevance in immune metabolism.[^9^, ^10^] Notably, we discovered that both UNC0638 and UNC0737 reduced IgG secretion in an unbiased phenotypic screen using human peripheral blood mononuclear cells (PBMC).^[23]^ These data hinted at a possible role for CHKA in this context, which prompted us to broadly survey potential CHKA‐dependent effects in clinically relevant primary human cell‐based tissue and disease models. We profiled UNC0638 and UNC0737 alongside the orthogonal CHKA inhibitors V‐11‐0711 and ARIAD against the BioMAP Diversity PLUS panel (Figures S7).^[24]^ This set comprises 148 distinct biomarkers across 12 systems, including panels for systemic immune responses. By comparing the overall responses across all biomarkers, both UNC0638 and UNC0737 clustered together with the known CHKA inhibitors V‐11‐0711 and ARIAD, whereas the CHKA‐inactive G9a/GLP inhibitor A‐366 did not exhibit any overlap (Figure 4A). The strongest biomarker changes were observed in the PBMC panel where UNC0638 and UNC0737 showed significant effects on IgG secretion, consistent with previous results (Figure 4B). We further confirmed dose‐dependent reduction of IgG secretion by UNC0638 and UNC0737 using PBMCs from healthy human blood donors (Figure 4C). The decrease in IgG secretion was accompanied by a reduction in stimulation‐induced B cell maturation into CD27^+^IgD^−^ memory CD19^+^ B cells (Figure 4D). Notably, flow cytometry analysis indicated that cell viability was not markedly affected by the inhibitors (Figure 4E). To confirm CHKA target engagement in this context, we also performed competitive chemoproteomics with healthy donor PBMC cell extracts which identified CHKA as the sole shared target of UNC0638 and UNC0737 in these cells (Figure 4F, Figure S8). Collectively, these data support the notion that small molecule CHKA inhibitors could present an attractive opportunity to modulate phospholipid metabolism in the context of immune‐related disorders.

In this study, we investigated the phenotypic and molecular target profiles of the G9a/GLP chemical probe UNC0638 and its companion negative control UNC0737 in human cells. Interestingly, we found that both UNC0638 and UNC0737 exhibited more pronounced effects on cancer cell viability than the chemically distinct G9a/GLP inhibitor A‐366. These results strongly supported the previously purported notion that UNC0638 may have additional off‐target(s) conferring the observed effect on cancer cell growth. Our comprehensive phenotypic, functional, biophysical as well as structural data conclusively establish both UNC0638 as well as its negative control UNC0737 as potent and cell active CHKA inhibitors. Since suppression of CHKA activity has previously been linked to impaired cancer cell proliferation, our data may therefore, at least in part, explain the antiproliferative effects observed for UNC0638 and UNC0737 by us and others.^[7]^ To uncover the direct molecular targets of UNC0638 and UNC0737, we performed both affinity‐based chemoproteomics as well as PISA thermal profiling which consistently identified CHKA as a shared target for both compounds underscoring the power of proteomic approaches to identify unforeseen and outside‐of‐class off‐targets.[^25^, ^26^, ^27^, ^28^] Notably, CHKA is not included in standard drug off‐target screening panels, and to our knowledge, not even covered by standard kinase‐specific chemoproteomic platforms. This observation is in line with previous reports describing challenges to identify chemical starting points for CHKA inhibitors using classic HTS approaches.^[6]^ Abnormal choline metabolism is a key feature of various metabolic and inflammatory disorders and has been linked to human cancers, including solid tumours and haematological malignancies.[^10^, ^29^] However, clinical progression of CHKA inhibitors has been hampered by toxicity, limited efficacy and/or poor pharmacokinetics of choline‐mimetic inhibitors.[^6^, ^30^] Compounds such as RSM‐932A (TCD‐717) have been inspired by the prototypic inhibitor HC3 containing a charged quaternary ammonium group which is considered unfavourable with regard to cell permeability and the potential to exhibit unspecific interactions (Figure S9).[^8^, ^31^] Currently, there is a significant lack of well‐annotated and widely available CHKA chemical probes. Here, were uncover a novel scaffold for CHKA inhibitors that is synthetically tractable and, in case of UNC0737, appears potent and selective based on our extensive phenotypic and mechanistic characterisation data. Our structural data revealed an intriguing binding mode showing that UNC0638 and UNC0737 both engage the substrate pocket targeted by choline‐mimetic inhibitors. Targeted metabolomic analysis in MCF7 cells confirmed that UNC0638 and UN0737 significantly reduced phospho‐choline levels and exhibited a dose‐dependent decrease in extracellular choline uptake compared to controls. It is worth noting that intracellular choline levels and sn‐glycerophosphocholine (GPC) were not significantly affected by treatment with either UNC0638 or UNC0737, suggesting that CHKA inhibition does not affect GPC contribution to intracellular choline levels. Similarly, we did not observe changes in betaine, the oxidation product of choline. We hypothesise that the reduced choline uptake upon CHKA inhibition may be due to a significantly slower choline phosphorylation rate. The fact that intracellular levels remain unaltered upon treatment with UNC0638 and UNC0737 might suggest a mechanism in which the intracellular levels of choline, its uptake from the extracellular space as well as its usage as a substrate for phosphocholine are highly intertwined and tightly regulated. Previous studies have suggested that some CHKA inhibitors might act on other targets in this pathway, such as choline transporters, which complicates interpretation of phenotypes.^[30]^ However, we did not observe any other significant targets for UNC0638 and UNC0737 in our chemoproteomic data. While the relevance of inhibiting CHKA catalytic activity in cancer remains to be investigated, our observations on IgG secretion in primary human immune assays align with reports suggesting potential alternative therapeutic applications for CHKA inhibitors in regulating immune signalling.[^9^, ^10^]

## Conclusion

The identification and validation of drug targets are pivotal steps in the development of novel therapeutic approaches. Small molecule tool compounds have the potential to be transformative assets in this context, however, extensive characterisation as well as considerate use of orthogonal chemotypes and negative control compounds are essential.[^32^, ^33^] We hope that our integrated workflow will inspire further efforts to characterize chemical probes, enhance their annotation, and provide an attractive complementary toolbox alongside genomic and transcriptomic approaches for mechanism of action studies.

## Data Deposition

The mass spectrometry proteomics data have been deposited to the ProteomeXchange Consortium via the PRIDE^[34]^ partner repository with the data set identifiers PXD056865 (chemical pulldowns), PXD057925 (PISA intact HepG2) and PXD058045 (PISA K562 lysate). Crystal structures have been deposited in the PDB (codes: 8BI5, 8BI6).

## Conflict of Interests

J.J. is a cofounder and equity shareholder in Cullgen, Inc., a scientific cofounder and scientific advisory board member of Onsero Therapeutics, Inc., and a consultant for Cullgen, Inc., EpiCypher, Inc., Accent Therapeutics, Inc, and Tavotek Biotherapeutics, Inc. The Jin laboratory received research funds from Celgene Corporation, Levo Therapeutics, Inc., Cullgen, Inc. and Cullinan Oncology, Inc.

1

## Supporting information

As a service to our authors and readers, this journal provides supporting information supplied by the authors. Such materials are peer reviewed and may be re‐organized for online delivery, but are not copy‐edited or typeset. Technical support issues arising from supporting information (other than missing files) should be addressed to the authors.

Supporting Information

Supporting Information

Supporting Information

Supporting Information

## Data Availability

Coordinates and structure factors of the inhibitor complexes are available in the Protein Data Bank (PDB) under accession codes 8BI6 and 8BI5.
