# Supplementary material for: Phenomics‐Based Discovery of Novel Orthosteric Choline Kinase Inhibitors
Source: Angew Chem Int Ed Engl. 2025 Jan 13;64(7):e202420149. doi: 10.1002/anie.202420149 (PMC11811597; doi:10.1002/anie.202420149)
Supplement: Supplementary file 1 — Supporting Information [file ANIE-64-e202420149-s003.pdf]

Angewandte Chemie
Eine Zeitschrift der Gesellschaft Deutscher Chemiker

Supporting Information

# Phenomics-Based Discovery of Novel Orthosteric Choline Kinase Inhibitors

*L. G. Bauer, J. A. Ward, L. Díaz-Sáez, Y. Sundström, T. Tolvanen, J. C. Alarcón Barrera, S. Kostidis, C. M. Rogers, I. Panagakou, U. Singh, E. M. Rothweiler, A. Gonzalez Orta, H. Ü. Kaniskan, J. Hu, J. Jin, S. Sievers, H. Waldmann, M. Giera, M. Sundström, L. Berg, K. V. M. Huber**

# Supporting information

## Phenomics-based Discovery of Novel Orthosteric Choline Kinase Inhibitors

Ludwig G. Bauer[1,2 +], Jennifer A. Ward[1,2 +], Laura Díaz-Sáez[1,2], Yvonne Sundström[3,4], Tuomas Tolvanen[3,5], Juan Carlos Alarcón Barrera[6], Sarantos Kostidis[6], Catherine M. Rogers[1,2], Ioanna Panagakou[1,2], Usha Singh [1,2], Elisabeth M. Rothweiler [1,2], Alejandro Gonzalez Orta[1,2], H. Ümit Kaniskan[7], Jianping Hu[7], Jian Jin[7], Sonja Sievers[8], Herbert Waldmann[9], Martin Giera[6], Michael Sundström[3,4], Louise Berg[3,4], Kilian V. M. Huber*[1,2]

[1]Centre for Medicines Discovery, Nuffield Department of Medicine, University of Oxford, Oxford, UK

[2]Target Discovery Institute, Nuffield Department of Medicine, University of Oxford, Oxford, UK

[3]Structural Genomics Consortium, Karolinska Institutet, Solna, Sweden

[4]Department of Medicine Solna, Karolinska Institutet and Karolinska University Hospital, Stockholm, Sweden

[5]Pelago Bioscience AB, Solna, Sweden

[6]Center for Proteomics and Metabolomics, Leiden University Medical Center, Leiden, The Netherlands

[7]Mount Sinai Center for Therapeutics Discovery, Departments of Pharmacological Sciences, Oncological Sciences and Neuroscience, Tisch Cancer Institute, Icahn School of Medicine at Mount Sinai, New York, NY 10029, USA

[8]Compound Management and Screening Center at the Max Planck Institute of Molecular Physiology, Dortmund, Germany

[9]Department of Chemical Biology, Max Planck Institute of Molecular Physiology, Dortmund, Germany

[+]These authors contributed equally

*Correspondence:
kilian.huber@cmd.ox.ac.uk

## Table of Contents

**Methods**…………………………………………………………………………………………………………………………...S3-12

**Supplementary Figures and Tables**……………………………………………………………………………....S13-20

**Figure S1**: UNC0737 impacts cell viability to similar extent as UNC0638…………………………………….....S13

**Figure S2**: Chemical structure of UNC0965…………………………………………………………………………......S13

**Figure S3**: Western blot of elution fractions after chemical pulldown with UNC0965 in MCF7 cell extract pre-incubated with indicated compounds (20 µM) or DMSO................................................S14

**Figure S4**: PISA thermal profiling results in K562 cell lysates............................................................S14

**Figure S5**: UNC0638 and UNC0737 reduce choline metabolism in MCF7 cells..................................S15

**Figure S6**: Biophysical assay results.....................................................................................S16

**Table S1**: Crystallography statistics.....................................................................................S17

**Figure S7**: BioMAP biomarker profiles of A) UNC0638 B) UNC0737 C) V-11-0711 D) ARIAD.........S18-19

**Figure S8**: Affinity-based chemoproteomics using UNC0965 functionalised affinity matrix and 20 µM UNC0638 or UNC0737 as competitors in PBMC cell extract..............................................................S19

**Figure S9:** Chemical structures of HC3 and RSM-932A…………………………………………………………………….S20

**References**..........................................................................................................................S21

## Methods

### Reagents

A-366 and UNC0638 were purchased from Tocris. ARIAD and V-11-07-11 were purchased from Wuxi Apptec. UNC0737 and UNC0965 were synthesised as previously described.[1] Hemicholinium-3 and pyruvate kinase/lactic dehydrogenase enzymes were purchased from Sigma Aldrich. Aqueous and organic solvents were MS-grade and acquired by Sigma Aldrich unless stated otherwise.

### Cell Culture

MDA-MB-231, MCF7 and HEK293 cells were cultured in DMEM (Gibco, #11965092) supplemented with 10% FBS. K562 were cultured in IMDM (Gibco, #12440053) supplemented with 10% FBS. HepG2 were cultured in MEM (Gibco, #11095080) supplemented with 10 % FBS. K562 were cultured in IMDM (Gibco, #12589059) supplemented with 10 % FBS and 1x GlutaMAX (Gibco, #35050061). U2OS were cultured in McCoy's 5A (Sigma, M8403) supplemented with 10 % FBS and 1x GlutaMAX (Gibco, #35050061). Cells were maintained at 37 °C in a humidified 5% $CO_2$ atmosphere. Peripheral blood mononuclear cells (PBMC) were isolated from healthy blood donors by density centrifugation.

### Cytotoxicity Assessment

MDA-MB-231, MCF7 or HEK293 cells ($7.5 \times 10^4$ cells/mL) were each plated into x1 white 384-well polystyrene plate (Greiner Bio-One Ltd). A-366, UNC0628 and UNC0737 were directly added to each well of the cell line plates, at final concentrations 0-50 µM and incubated for 72 h at 37 °C/ 5% $CO_2$. Cell Titer-Glo® reagent was added to each well and total luminescence readings were performed within 15 min using the PHERAstar FSX (BMG Labtech). The cell viability results were normalised to the DMSO control and plotted using the sigmoidal dose-response (variable slope) equation in GraphPad Prism.

### Chemical Proteomics Drug Pulldown

For lysate generation MCF7 cells were grown until approximately 80% confluency before being pelleted and washed with PBS. Cell pellets were subsequently lysed by addition of Buffer A (50 mM Tris pH 7.5, 0. 8% *v/v* NP-40, 5% *v/v* glycerol, 1.5 mM $MgCl_2$, 100 mM NaCl, 25 mM NaF, 1 mM $Na_3VO_4$, 1 mM PMSF, 1 mM DTT, 10 µg $mL^{-1}$ TLCK, 1 µg $mL^{-1}$ Leupeptin, 1 µg $mL^{-1}$ Aprotinin, 1 µg $mL^{-1}$ soy bean trypsin) as previously described.[2]

For each pulldown condition, UNC0965 was coupled to 100 µL of UltraLink Immobilized Streptavidin Plus bead slurry (50% in isopropanol, GE Healthcare) to generate the affinity matrix as previously

described.[2] Lysates were precleared with bead slurry (100 µL) for 30 min at 4 °C and then treated with UNC0638 or UNC0737 (20 µM) or DMSO control for 30 min at 4 °C followed by incubation with affinity matrix for 2 h at 4 °C. Affinity matrices were washed with buffer A (5 mL) and proteins eluted with 100 µL of 2x Laemmli sample buffer, 100 mM DTT, in PBS and heating for 10 min at 90 °C. Eluents (70 µL) were diluted in 0.1 M Tris (pH 7.8) to 200 µL, reduced with 5 mM DTT for 30 min at RT, and alkylated with 20 mM iodoacetamide for 30 min at RT in the dark. Proteins were precipitated by sequential addition of MeOH (600 µL), $CHCl_3$ (150 µL) and $H_2O$ (450 µL), pelleted (17,000 × *g*, 5 min), washed with further MeOH (2 × 600 µL), and re-pelleted. Air dried pellets were resuspended in 50 µL 6 M urea (pH 7.8) by vortexing and then diluted with 250 µL $H_2O$ before adding 1 µg trypsin for overnight incubation at 37 °C. The digests were acidified with FA (1% *v/v* final concentration), desalted using SOLA HRP SPE Cartridges (Thermo Fisher), eluted with 69% *v/v* MeCN, 0.1% *v/v* FA in $H_2O$ (600 µL) and dried *in vacuo*. Dried peptides were stored at -20 °C before resuspension in 2% *v/v* MeCN, 0.1% *v/v* FA in $H_2O$ (20 µL) for LC-MS/MS analysis.

**LC-MS/MS Data Acquisition**

Digested samples were analysed by nano-UPLC–MS/MS using a Dionex Ultimate 3000 nano UPLC fitted with an EASY spray column (75 µm × 500 mm, 2 µm particle size, Thermo Scientific), coupled to an Orbitrap Q Exactive instrument. A 60 min gradient of 0.1% (*v/v*) formic acid in 5 % (*v/v*) DMSO to 0.1% (*v/v*) formic acid with 35% (*v/v*) acetonitrile in 5 % (*v/v*) DMSO at a flow rate of 250 nL $min^{-1}$ was used. The instrument was operated in a data-dependant mode, with survey scans acquired at a resolution of 70,000 at 200 m/z and the 15 most abundant precursors selected for HCD fragmentation with an AGC target of 1 x $10^5$ ions.

**LC-MS/MS Data Analysis**

Raw data was processed using MaxQuant[3] version 2.0.1.0 and the reference complete human proteome FASTA file (UniProt, downloaded 05.08.21). Label Free Quantification (LFQ) and Match Between Runs were selected; Cysteine carbamidomethylation was selected as a fixed modification, and methionine oxidation and asparagine deamidation as variable modifications. Default settings for identification and quantification were used. Specifically, a minimum peptide length of 7, a maximum of 2 missed cleavage sites, and a maximum of 5 labelled amino acids per peptide were allowed. Through selection of the 'trypsin/P' general setting, peptide bond cleavage at arginine or lysine (followed by any amino acid) was considered during *in silico* digest of the reference proteome. The allowed precursor and fragment ion mass tolerances were 4.5 ppm and 20 ppm, respectively. Peptides and proteins were identified utilizing a 0.01 false discovery rate, with "unique and razor peptides"

mode selected for both identification and quantification of proteins (razor peptides are uniquely assigned to protein groups and not to individual proteins). At least 1 razor + unique peptides were required for valid quantification. Processed data was further analysed using Perseus version 1.6.15.0.[4] Peptides categorized by MaxQuant as 'potential contaminants', 'only identified by site' or 'reverse' were filtered, and the LFQ intensities $\log_2$ transformed. Experimental replicates were grouped, and only 2 out 6 missing values were allowed. Protein groups were additionally filtered by >2 unique peptides. Missing values were imputed from a normal distribution using default settings, and the data distribution visually inspected to ensure that a normal distribution was maintained. Data were visualised in volcano plots using R studio version 4.1.1. Significantly competed targets were further analysed in STRING (http://string-db.org) and protein interaction networks generated. Basic STRING settings were used for network analysis of enriched proteins. Specifically, network edges represent confidence in interaction. Line thickness indicates the strength of data support with a minimum required interaction score of 0.400. All active interaction sources (Textmining, Experiments, Databases, Co-expression, Neighborhood, Gene Fusion, Co-occurrence) were considered.

**Western Blotting**

Proteins were separated by SDS-PAGE and transferred to nitrocellulose blotting membranes. Membranes were blocked with blocking buffer (2.5% (*m/v*) BLOT-QuickBlocker (Merck) in PBST (Phosphate-buffered saline with Tween: 4.3 mM $Na_2HPO_4$, 1.47 mM $KH_2PO_4$, 137 mM NaCl, 2.7 mM KCl, 0.05% (*v/v*) Tween 20) before probing the indicated antibodies. Blots were visualised on a LiCOR Odysse, and bands quantified relative to loading controls using Image Studio Software (LiCOR). Primary antibodies: anti-CHKA (Proteintech, 13520-1-AP), anti-EHMT2 (Bethyl Laboratories, A300-933A-T). Secondary antibodies: Goat anti-Mouse IRDye® 800CW (Abcam, ab216772), Goat Anti-Rabbit IRDye® 680RD (Abcam, ab216777).

**Protein Expression and Purification**

pET28a-LIC-$His_6$-CHKA (Addgene #25515) was transformed into *E. coli* BL21(DE3) cells. The cells were cultured at 37 °C in the presence of 50 µg/mL of kanamycin in TB media until an OD of 0.6 was reached. Induction was achieved with IPTG (200 µM final concentration) and overnight incubation at 18 °C. The cells were harvested by centrifugation (35,000 × *g*, 15 min) and the resulting cell pellet lysed by French press in 30 mL Buffer A (20 mM HEPES, 500 mM NaCl, 5% glycerol, 20 mM imidazole, 0.5% TCEP, pH 7.5) supplemented with Protease Inhibitor Cocktail Set III, EDTA-Free (Merck), and benzonase. The lysate was spun at 35,000 × *g* for 1 h at 4 °C before being purified using a HisTrap FF 5 mL column (Cytiva) equilibrated in Buffer A. $His_6$-tagged protein was eluted with Buffer B (20 mM HEPES, 500

mM NaCl, 5% glycerol, 500 mM imidazole, 0.5% TCEP, pH 7.5), the eluent was concentrated, and further purified by size exclusion chromatography using a HiLoad 16/600 Superdex S200 column (Cytiva) pre-equilibrated in Buffer C (20 mM HEPES, 150 mM NaCl, pH 7.5). The resulting protein purity was assessed by SDS-PAGE, and protein identity was confirmed by tryptic digest analysis. Protein concentration was determined to be 35.5 mg/mL, and was calculated using a theoretical molar extinction coefficient of 58830 $M^{-1}cm^{-1}$ as determined by ProtParam.[5]

**Isothermal Calorimetry (ITC)**

ITC experiments were performed in a buffer containing 20 mM HEPES pH 7.5 and 200 mM NaCl, using an VP-ITC microcalorimeter (MicroCal) at 15 °C. The protein concentration was 200 µM and compounds were tested at 20 µM. The titrations were conducted using an initial injection of 4 µL followed by further 29 injections of 8 µL each. Thermodynamic parameters were calculated using $\Delta G = \Delta H - T\Delta S = -RT \ln KB$, where ΔG, ΔH, and ΔS are the changes in free energy, enthalpy, and entropy of binding, respectively. Single binding models were employed to fit the data.

**Differential Scanning Fluorimetry (DSF)**

DSF buffer (20 mM HEPES, 150 mM NaCl, 0.5 mM TCEP, pH 7.5) containing 5x SYPRO Orange (Thermo Fisher Scientific) and CHKA (2 µM) was incubated with 10 µM compound at 2% DMSO final concentration in a 96-well semi-skirted real time PCR plate. Fluorescence was measured in each well over a 40-80 °C gradient using a Stratagen MX3005p qPCR instrument (Agilent Technologies).

**Crystallography**

Purified CHKA protein at 33 mg/mL was initially incubated for 1 h with 1:3 protein:compound molar ratios for both UNC0638 and UNC0737. Crystallisation plates were set up with 3 different protein:reservoir ratios (2:1, 1:1, 1:2) using different commercial crystallisation screens, and incubated at 20 °C. Crystals that resulted in the final structures were obtained. In the case of UNC0638, crystals grew in a reservoir condition containing 30% PEG smear low molecular weight (Molecular dimensions PEG mixture), and 0.1 M MES pH 6.5. UNC0737 containing crystals grew from drops with a reservoir condition of 22.5% PEG smear low molecular weight, 0.1 M MES pH 6.5 and 10% (*v/v*) isopropanol. Crystals were cryoprotected with 25% ethylene glycol, flash frozen with liquid nitrogen, and data collection was carried out at the Diamond Light Source beamline i03. Data were indexed and integrated using the software autoPROC,[6] then scaled and analysed with AIMLESS.[7] The previously characterised CHKA structure (PDB 5EQP) was used as the search model in PHASER.[8] Refinement was

carried out using REFMAC[9] and COOT,[10] and concluded when no improvements resulted for $R_{\text{free}}$ and $R_{\text{work}}$ values, and when density maps indicated no further changes were justified.

**Choline Coupled Enzymatic Assay**

CHKA (120 ng, 24 µg/ml) was arrayed (5 µL) into a 96 well semi-skirted real time PCR plate. 4-fold dilution series and vehicle controls (0.5% DMSO) were prepared in a separate plate. A further DMSO control was included for use with a no protein well control. The dilutions (17 µL) were mixed with reaction buffer (136 µL, see Table below) and arrayed (45 µL) onto the CHKA-containing PCR plate. After mixing, bubbles were removed by centrifugation (1000 × *g*, 1 min) and absorbance read at 340 nM on a PheraSTAR FSX (BMG Labtech), reading every minute for 10 min. Non-linear curve fitting and graph generation was performed using Graphpad Prism 8 software.

Components of the assay reaction buffer.

| **Component** | **Stock concentration** | **Volume per buffer mix (µL)** | **Final reaction concentration** |
|---|---|---|---|
| TRIS pH 7.5 | 1 M | 360 | 100 mM |
| KCl | 1 M | 360 | 100 mM |
| $MgCl_2$ | 100 mM | 360 | 10 mM |
| PEP | 5 mM | 360 | 500 µM |
| choline chloride | 966 µM | 360 | 96.6 µM |
| NADH | 2.5 mM | 360 | 250 µM |
| PK/LDH enzyme mix | 900-1400/600/1000 U/mL | 360 | - |
| ATP | 4.5 mM | 360 | 450 µM |

**CETSA Methods**

Experiments were performed as described earlier[11-12] with few modifications. In short, 3 replicates of intact HepG2 cells were treated with 30 µM UNC0638, UNC373 or corresponding volume of DMSO in medium-free experimental buffer for 60 min at 37 °C with end-over-end rotation. For lysates: Washed K562 cells were resuspended in medium-free experimental buffer and lysed by three freeze-thaw cycles. Lysate was centrifugated 20,000 × *g* for 20 min at 4 °C to remove cell debris. The cleared lysate was treated with 30 µM UNC0638, UNC0373 or corresponding volume of DMSO for 15 min at RT with end-over-end rotation. Each treated cell suspension or lysate was divided into 12 aliquots and subjected to a 12-step heat gradient from 44 °C to 64 °C. All the temperature points were pooled together to form nine individual samples. Soluble fraction was separated from precipitated proteins

by centrifugation at 30,000 × *g* for 20 min at 4 °C. The soluble fractions were collected to fresh tubes and further processed to be analysed with mass spectrometry. Sample handling, TMT labelling, LC-MS/MS analysis, protein identification and data analysis was performed as described earlier.[12]

**NMR Choline Metabolite Analysis**

Sample preparation for the quantification of metabolites by nuclear magnetic resonance (NMR) spectroscopy was performed as described previously.[13] Briefly, 0.3 mL of cell culture medium (conditioned medium) was collected from each sample and mixed with 2 volumes of pre-chilled methanol (< -70 °C), vortexed for 30 s and placed on dry ice for at least 30 min. Following centrifugation for 20 min at 18100 × *g* and 0 °C, the supernatant extract of culture medium was dried and stored until NMR analysis. The remaining culture medium from each sample was discarded and cells were washed once with PBS (37 °C), and quenched with liquid nitrogen. Polar metabolites were extracted with 1.4 mL of an ice-cold solution of 67.5:7.5:25 (*v/v/v*) of methanol/chloroform/water. Samples were centrifuged at 18100 × *g* for 15 min at 0 °C and the supernatant consisted of intracellular polar metabolites was collected and dried under nitrogen gas stream. The remaining cell pellets were used for protein mass quantification using the Pierce™ BCA Protein Assay Kit (Thermo Scientific™). Samples from both cell culture medium and cell extracts were reconstituted in 220 µL of 50 mM phosphate buffer (pH 7.4) solution in deuterated water containing 0.05 mM trimethylsilyl propionic-*d4*-sodium salt (TSP-*d4*, Cambridge Isotope Laboratories, Inc.) as internal standard used for NMR referencing and quantification. One NMR experiment (pulse sequence: *noesygppr1d*; Bruker Biospin) was collected for each sample in a 14.1 T (600 MHz for $^{1}$H) Bruker Avance Neo NMR. All recorded NMR spectra were imported in Chenomx NMR suite 9.0 (Chenomx NMR suite, v9.0) for the quantification of choline, phosphocholine sn-glycero-phosphocholine (GPC) and betaine. Quantitative data (nmoles or µM) were then normalized to the amount of total protein mass (mg) of each sample.

**B-Cell Assay**

PBMCs from seven healthy donors, were tested in the B cell assay. PBMCs were isolated and stimulated with IL-4, IL-10, IL-21, ODN2006 and sCD40L for 6 days to mature the B cells and induce secretion of IgG (for details, see [14]). After six days of cell cultures, culture supernatants were examined for concentration of IgG by a commercial ELISA kit (Mabtech, Sweden) and for cytokines by Bio-Rad's 37-Plex panel #171AL001M. The cells were analysed by flow cytometry including cellular markers for B cell maturation (CD19, CD27, CD38, surface IgD) as well as viability. FlowJo software (BD Biosciences) was used for data analysis. The blood was obtained under an approved ethical review from the Regional Ethical Review Board in Stockholm, approval number 2015/2001-31/2. The donors gave their consent to participate after written and oral information.

**Cell painting assay**

The described assay follows closely the method described by Bray et al.[15]

Initially, 5 µL U2OS medium were added to each well of a 384-well plate (Revvity Phenoplate 384). Subsequently, U2OS cell were seeded with a density of 1600 cells per well in 20 µL medium. The plate was incubated for 10 min at the ambient temperature, followed by an additional 4 h incubation (37 °C, 5% CO2). Compound treatment was performed with the Echo 520 acoustic dispenser (Beckman-Coulter) followed by incubation for 20 h (37 °C, 5% CO2). Subsequently, mitochondria were stained with Mito Tracker Deep Red (Thermo Fisher Scientific, Cat. No. M22426). The Mito Tracker Deep Red stock solution (1 mM) was diluted to a final concentration of 100 nM in prewarmed medium. The medium was removed from the plate leaving 10 µL residual volume and 25 µL of the Mito Tracker solution were added to each well. The plate was incubated for 30 min in darkness (37 °C, 5% CO2). To fix the cells 7 µL of 18.5 % formaldehyde in PBS were added, resulting in a final formaldehyde concentration of 3.7 %. Subsequently, the plate was incubated for another 20 min in darkness (RT) and washed three times with 70 µL of PBS. (Agilent Washer Elx405). Cells were permeabilized by addition of 25 µL 0.1% Triton X-100 to each well, followed by 15 min incubation (RT) in darkness. The cells were washed three times with PBS leaving a final volume of 10 µL. To each well 25 µL of a staining solution were added, which contains 1% BSA, 5 µL/ml Phalloidin (Alexa594 conjugate, Thermo Fisher Scientific, A12381), 25 µg/ml Concanavalin A (Alexa488 conjugate, Thermo Fisher Scientific, Cat. No. C11252), 5 µg/ml Hoechst 33342 (Sigma, Cat. No. B2261-25mg), 1.5 µg/ml WGA-Alexa594 conjugate (Thermo Fisher Scientific, Cat. No. W11262) and 1.5 µM SYTO 14 solution (Thermo Fisher Scientific, Cat. No. S7576). The plate is incubated for 30 min (RT) in darkness and washed three times with 70 µl PBS. After the final washing step, the PBS was not aspirated. The plates were sealed and centrifuged for 1 min at 50 x g.

The plates were prepared in triplicates with shifted layouts to reduce plate effects and imaged using a Micro XL High-Content Screening System (Molecular Devices) in 5 channels (DAPI: Ex350-400/ Em410-480; FITC: Ex470-500/ Em510-540; Spectrum Gold: Ex520-545/ Em560-585; TxRed: Ex535-585/ Em600-650; Cy5: Ex605-650/ Em670-715) with 9 sites per well and 20x magnification (binning 2).

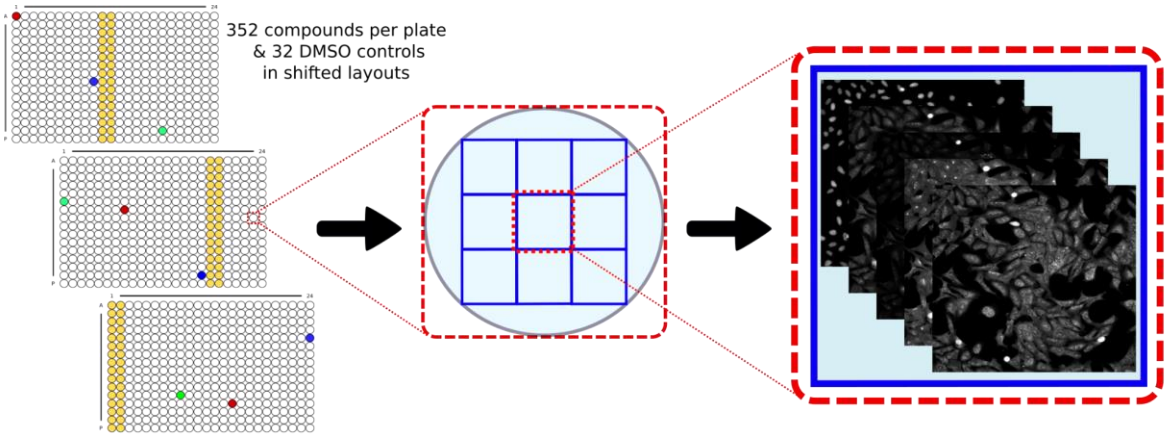


The generated images were processed with the *CellProfiler* package (https://cellprofiler.org/, version 3.0.0) on a computing cluster of the Max Planck Society to extract 1716 cell features per microscope site. The data was then further aggregated as medians per well (9 sites -> 1 well), then over the three replicates.

Further analysis was performed with custom *Python* (https://www.python.org/) scripts using the *Pandas* (https://pandas.pydata.org/) and *Dask* (https://dask.org/) data processing libraries as well as the *Scientific Python* (https://scipy.org/) package (separate publication to follow).

From the total set of 1716 features, a subset of highly reproducible and robust features was determined using the procedure described by Woehrmann et al.[16] in the following way: Two biological repeats of one plate containing reference compounds were analysed. For every feature, its full profile over each whole plate was calculated. If the profiles from the two repeats showed a similarity >= 0.8 (see below), the feature was added to the set. This procedure resulted in a set of 579 robust features out of the total of 1716 that was used for all further analyses.

The phenotypic profiles were compiled from the Z-scores of all individual cellular features, where the Z-score is a measure of how far away a data point is from a median value.

Specifically, Z-scores of test compounds were calculated relative to the Median of DMSO controls. Thus, the Z-score of a test compound defines how many MADs (Median Absolute Deviations) the measured value is away from the Median of the controls as illustrated by the following formula:

$$z - score = \frac{value_{meas.} - Median_{Controls}}{MAD_{Controls}}$$

The phenotypic compound profile is then determined as the list of Z-scores of all features for one compound.

In addition to the phenotypic profile, an induction value was determined for each compound as the fraction of significantly changed features, in percent:

$$Induction\ [\%] = \frac{number\ of\ features\ with\ abs.\ \ values\ >\ 3}{total\ number\ of\ features}$$

Similarities of phenotypic profiles (termed *Biosimilarity*) were calculated from the correlation distances (CD) between two profiles (https://docs.scipy.org/doc/scipy/reference/generated/scipy.spatial.distance.correlation.html):

$$CD = 1 - \frac{(u - \bar{u}) \cdot (v - \bar{v})}{\|(u - \bar{u})\|_2 \|(v - \bar{v})\|_2}$$

where $\bar{x}$ is the mean of the elements of $x$, $x \cdot y$ is the dot product of $x$ and $y$, and $\|x\|_2$is the Euclidean norm of $x$:

$$\|x\|_2 = \sqrt{x_1^2 + x_2^2 + \cdots + x_n^2}$$

The Biosimilarity is then defined as:

$$Biosimilarity = 1 - CD$$

Biosimilarity values smaller than 0 are set to 0 and the Biosimilarity is expressed in percent (0-100).
In addition to calculating biosimilarity between the full morphological profiles of two Cell painting measurements, Pahl et al. developed an approach to assign similarity to biological clusters by comparing sub-profiles.[17]
In essence, a set of 12 biological clusters was defined from Cell painting measurements with confirmed activity on these clusters. By considering only the features with similar values from the group of measurements for each cluster, a representative median profile was calculated for each cluster. These representative median profiles are of different length and shape for each cluster. By comparing the median cluster profiles to the matching sub-profiles of measured compounds, a biosimilarity to each cluster can be calculated. Furthermore, subprofiles can also be compared using the non-cluster features and not the cluster-defining features.

## Supplementary Figures and Tables

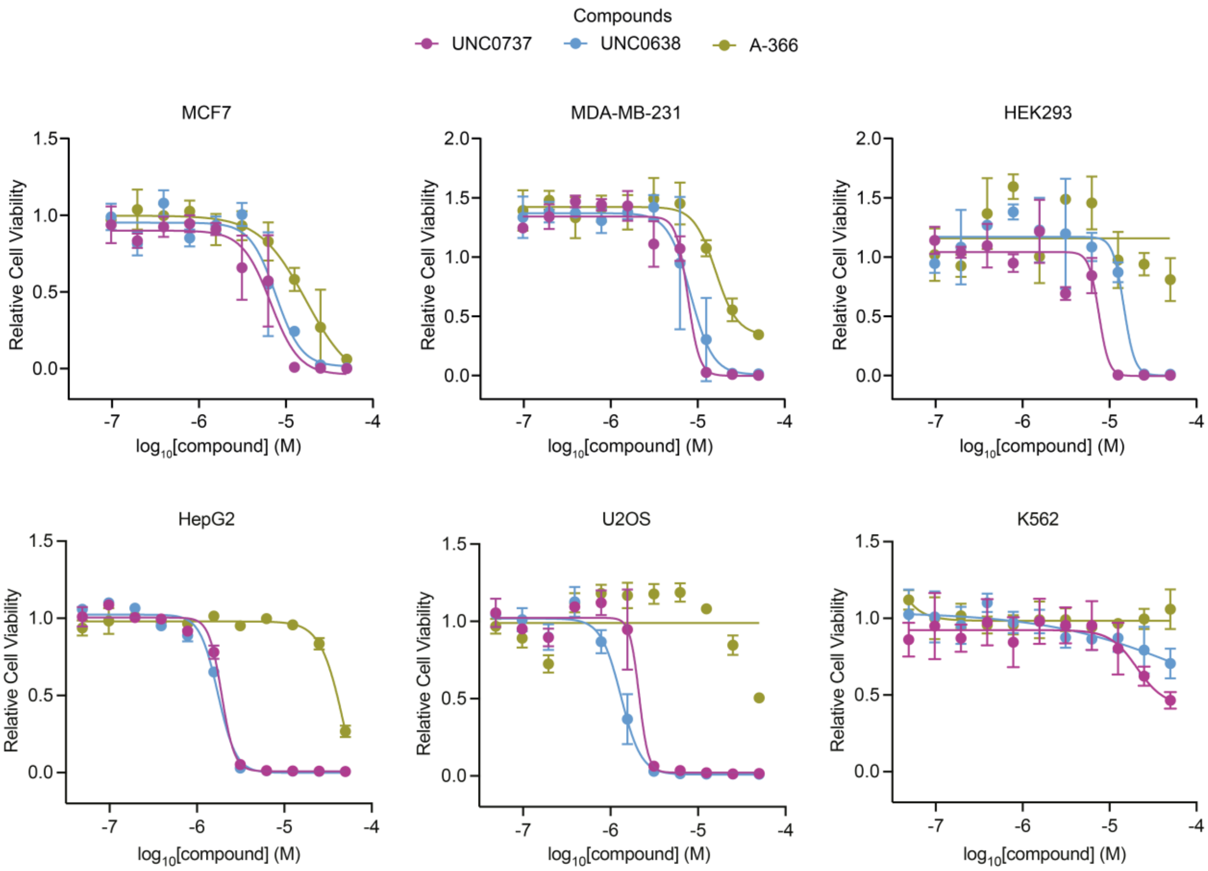


**Figure S1**: UNC0737 impacts cell viability to similar extent as UNC0638. A) Cell viability of HEK293, MDA-MB-231, HEK392, HepG2, U2OS and K562 cells following treatment with A-366, UNC0638 and UNC0737 after 72 h measured by Cell Titer Glo assay (n=3).

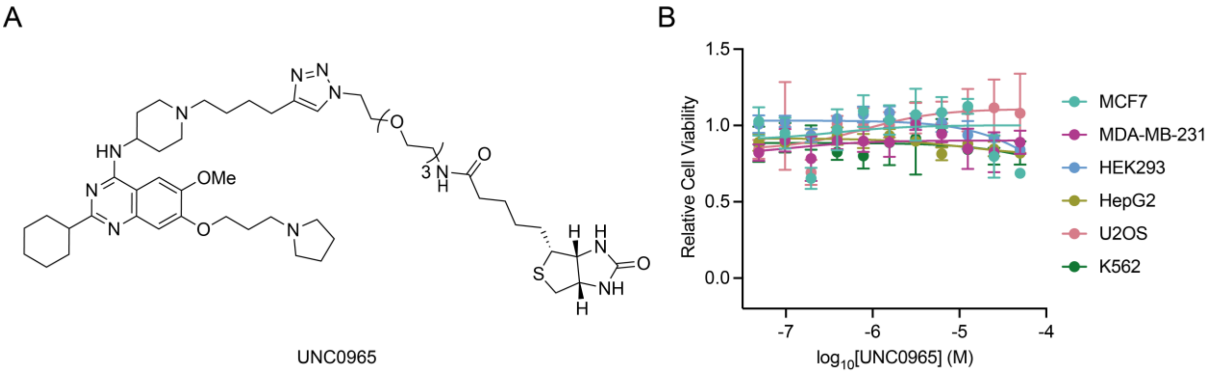


**Figure S2**: Chemical structure of UNC0965 (A) and cell viability of MCF7 cells following treatment with UNC0737 and UNC0965 (B). Cell viability upon UNC0965 treatment in indicated cell lines for 72 h (n = 3).

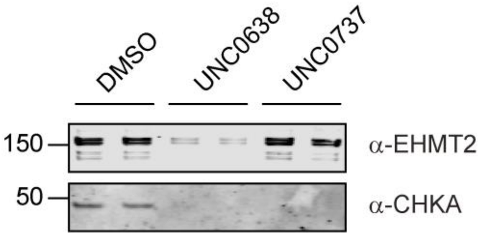


**Figure S3**: Western blot of elution fractions after chemical pulldown with UNC0965 in MCF7 cell extract pre-incubated with indicated compounds (20 µM) or DMSO.

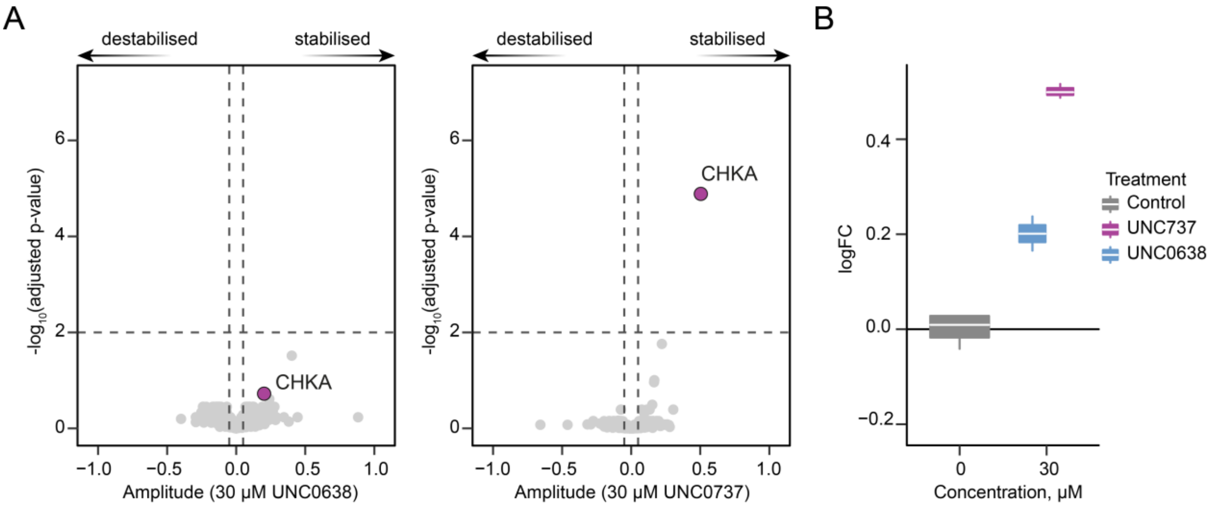


**Figure S4**: PISA thermal profiling results in K562 cell lysates. A) Volcano plots of UNC0638 and UNC0737 showing selective stabilisation of CHKA. Dashed lines represent amplitude > 0.05, < - 0.05 and $\log_{10}$(adjusted p-values) > 2 (two-sided two sample t-test, Benjamini-Hochberg corrected, n = 3). 6,924 proteins were identified. B) UNC0737 stabilises CHKA stronger than UNC0638.

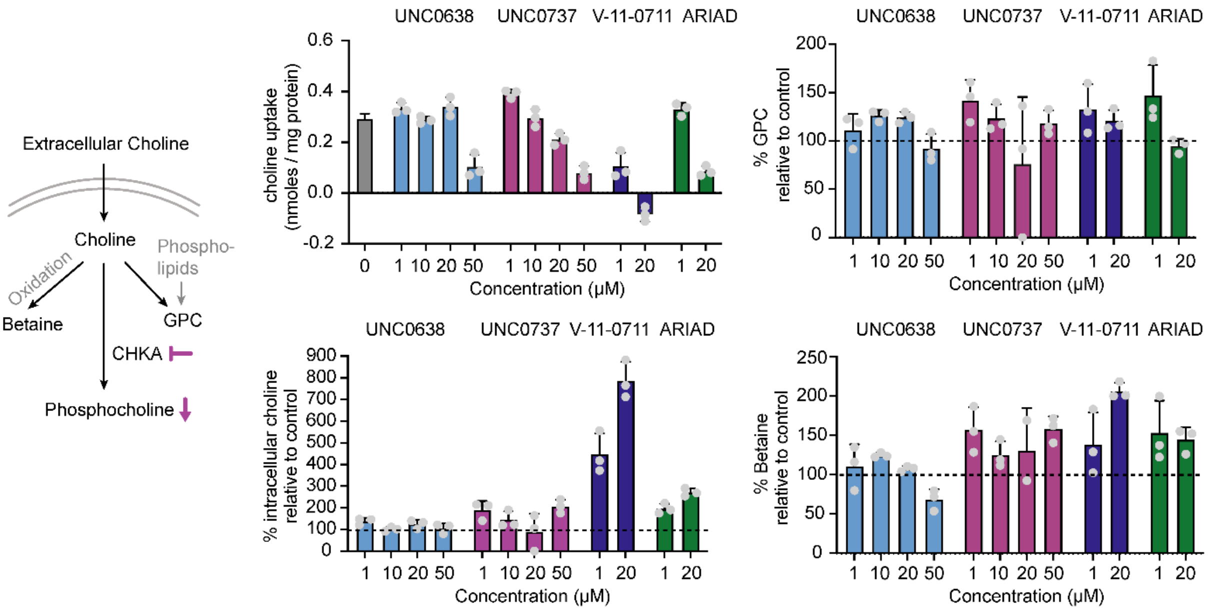


**Figure S5**: UNC0638 and UNC0737 reduce choline metabolism in MCF7 cells. NMR analysis of choline uptake, intracellular choline level, sn-glycero-3-phosphocholine (GPC) and betaine levels relative to vehicle control following incubation with indicated compounds. No change (100%) is marked with a dashed line (n =3).

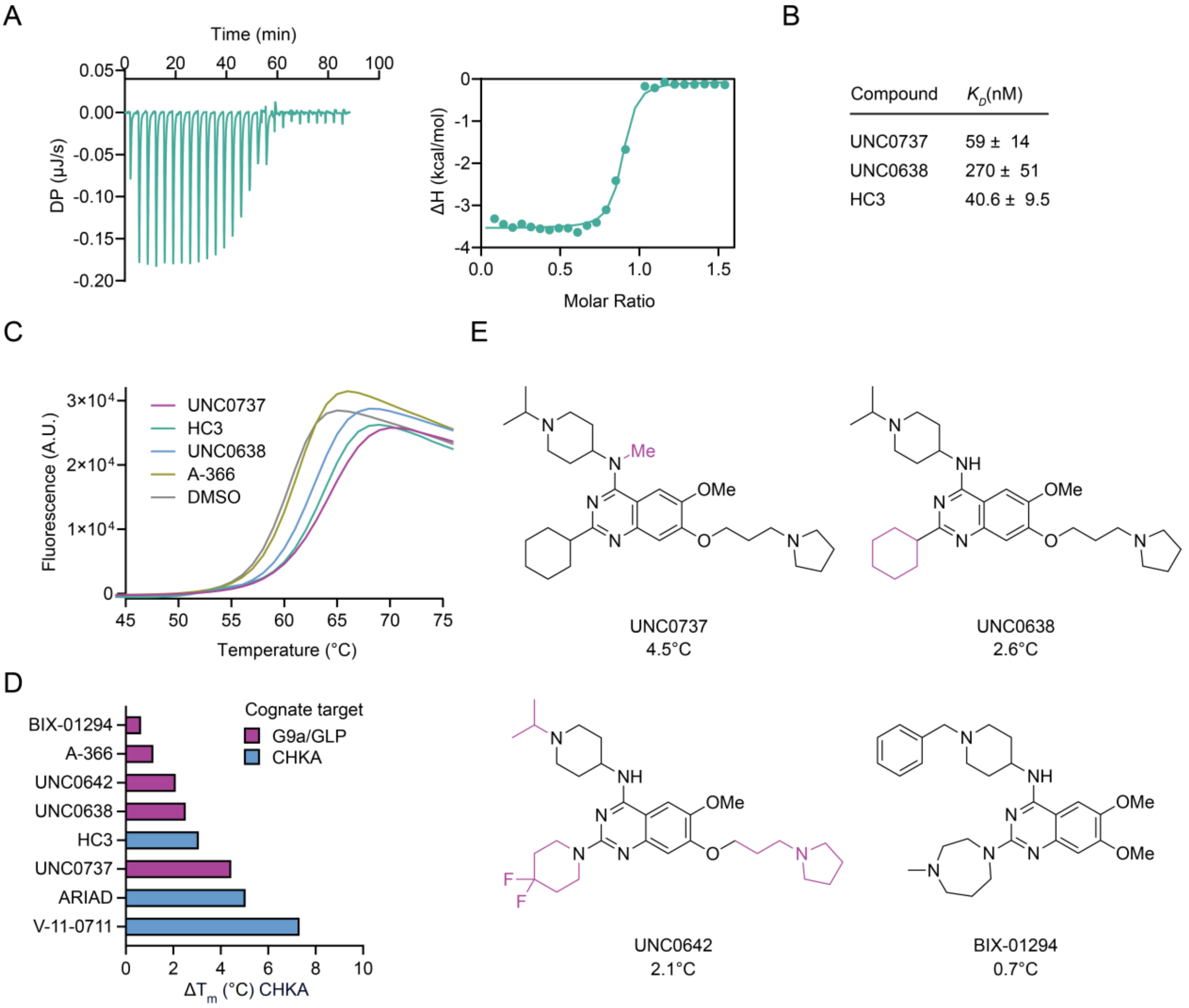


**Figure S6**: Biophysical assay results. A) ITC of HC3 against CHKA protein ($K_D$ = 40.6 ± 9.5 nM) (n = 1). B) Summary table of ITC results for indicated compounds against CHKA protein. C) DSF melting curves of 10 µM UNC0737, HC3, UNC0638, A-366 incubated with 2 µM CHKA protein. D) Bar graph of DSF results of 10 µM of indicated compounds incubated with 2 µM CHKA protein (n = 1). Melting temperature shifts ($\Delta T_m$) are expressed relative to DMSO control. E) Chemical structures and corresponding CHKA $\Delta T_m$ of UNC0737, UNC0638, UNC0642 and BIX-01294. Structural differences are marked in pink.

**Table S1**: Crystallography statistics. Values in parentheses refer to the highest resolution shell. [b] $R_{merge} = \sum_{hkl}\sum_i | I_i(hkl) - \langle I(hkl) \rangle | / \sum_{hkl}\sum_i I_i(hkl)$; where $I_i(hkl)$ is the intensity of the *i*th measurement of reflection *hkl* and <I(*hkl*)> is the mean value of $I_i(hkl)$ for all *i* measurements. [c] $R_{work} = \sum_{hkl} | |F_o| - |F_c| | / \sum |F_o|$, where $F_o$ is the observed structure factor and $F_c$ is the calculated structure factor. [d] $R_{free}$ is the same as $R_{work}$ except calculated with a subset, 5 %, of data that are excluded from the refinement calculations. [e] Diffraction Precision Index. [g] Protein atoms.

| **Structure**<br>**PDB code** | **CHKA:UNC0737**<br>**8BI5** | **CHKA:UNC0638**<br>**8BI6** |
|---|---|---|
| Space group | $P4_32_12$ | $P4_32_12$ |
| Wavelength (Å) | 0.976250 | 0.976250 |
| Unit cell dimensions<br>*a*, *b*, *c* (Å), α/β/γ (º) | 132.49 132.49 168.98<br>90.00 | 132.03 132.03 168.99<br>90.00 |
| Resolution range[a] (Å) | 48.25 – 2.50 | 48.40 – 2.40 |
| No. Reflections | 670428 | 763516 |
| Unique reflections | 51852 | 58985 |
| Completeness (%) | 100.0 (100.0) | 99.5 (99.4) |
| $R_{merge}$[b] | 0.061 (0.358) | 0.060 (0.422) |
| $CC_{1/2}$ | 0.999 (0.974) | 0.999 (0.815) |
| Redundancy | 12.9 (12.9) | 12.9 (13.5) |
| < I/σ(I)> | 26.9 (7.7) | §24.8 (5.8) |
| Wilson *B* ($Å^2$) | 46.92 | 47.32 |
| $R_{work}$[c]/$R_{free}$[d] | 0.1869 / 0.2128 | 0.1711/ 0.2100 |
| DPI[e] (Å) | 0.1965 | 0.1939 |
| Bond lengths (Å) /<br>angles[f] (º) | 0.0055 / 1.3545 | 0.0100 / 1.7249 |
| Average *B*-factors ($Å^2$)[g] | 56.43 | 58.58 |
| Ligand average *B*-factors ($Å^2$) | 58.76 | 91.00 (poorer density in some areas of the compound) |
| Ligands and ions | 2 UNC0737, 2 $Mg^{2+}$, 5 $Cl^-$, 3 $PO_4^{2-}$, 10 ethylene glycol | 2 UNC0638, 2 $Mg^{2+}$, 2 $Cl^-$, 4 $PO_4^{2-}$, 12 ethylene glycol, 2 polyethylene glycol, 2 isopropanol |
| Ramachandran analyses | | |
| Favoured regions (%) | 97.92 | 98.71 |
| Allowed regions (%) | 99.84 | 99.84 |

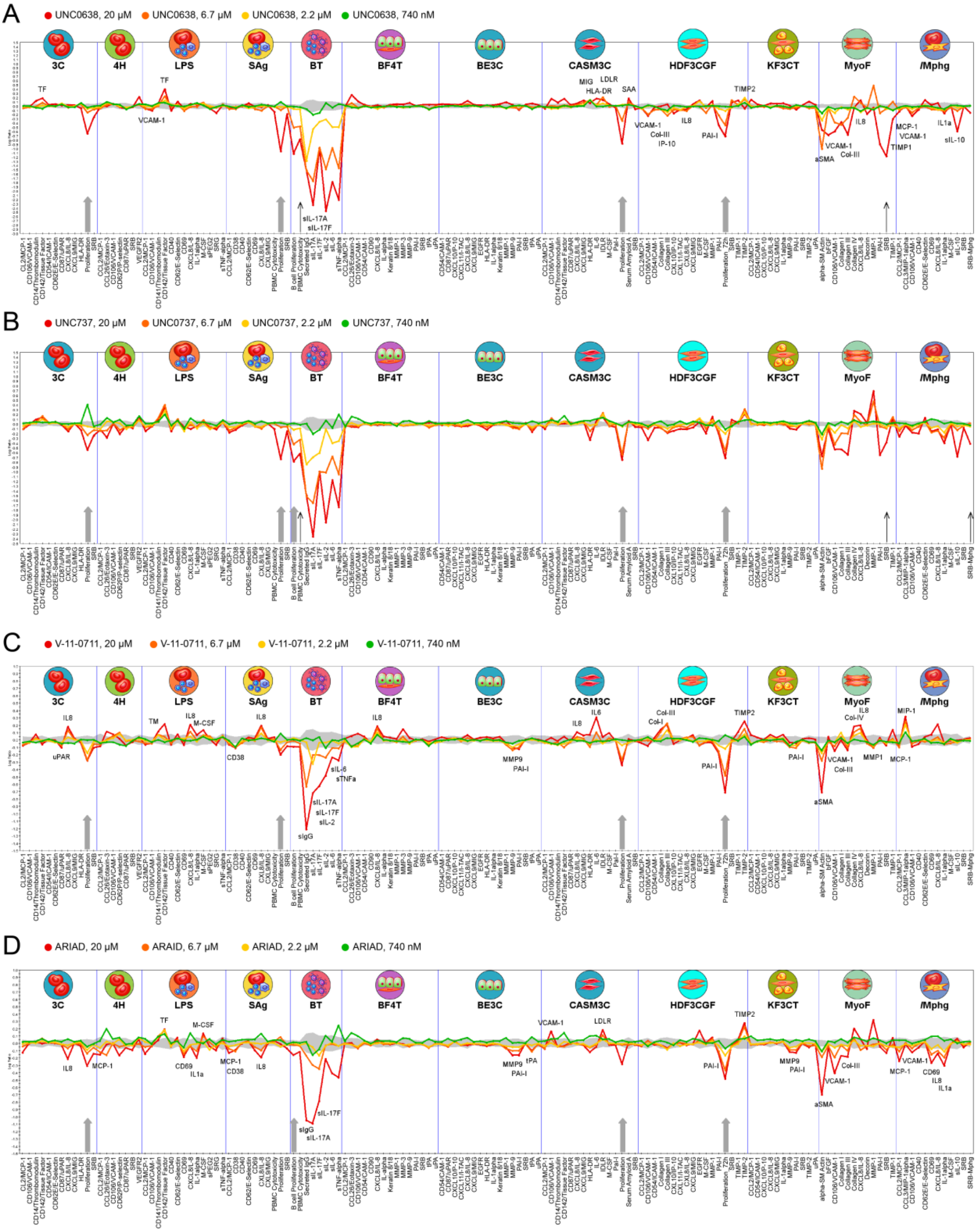


**Figure S7**: BioMAP biomarker profiles of A) UNC0638 B) UNC0737 C) V-11-0711 D) ARIAD. The x-axis lists the quantitative protein-based biomarker readouts measured in each system. The y-axis represents a log-transformed ratio of the biomarker readouts for the drug-treated sample (n = 1) over vehicle controls (n ≥ 6). The grey region around the y-axis represents the 95% significance envelope generated from historical vehicle controls. Biomarker activities are annotated when two or more

consecutive concentrations change in the same direction relative to vehicle controls, are outside of the significance envelope, and have at least one concentration with an effect size > 20% (|log10ratio| > 0.1). Biomarker key activities are described as modulated if these activities increase in some systems, but decrease in others. Cytotoxicity is indicated on the profile plot by a thin black arrow above the x-axis, and antiproliferative effects are indicated by a thick grey arrow. Cytotoxicity and antiproliferative arrows only require one concentration to meet the indicated threshold for profile annotation.

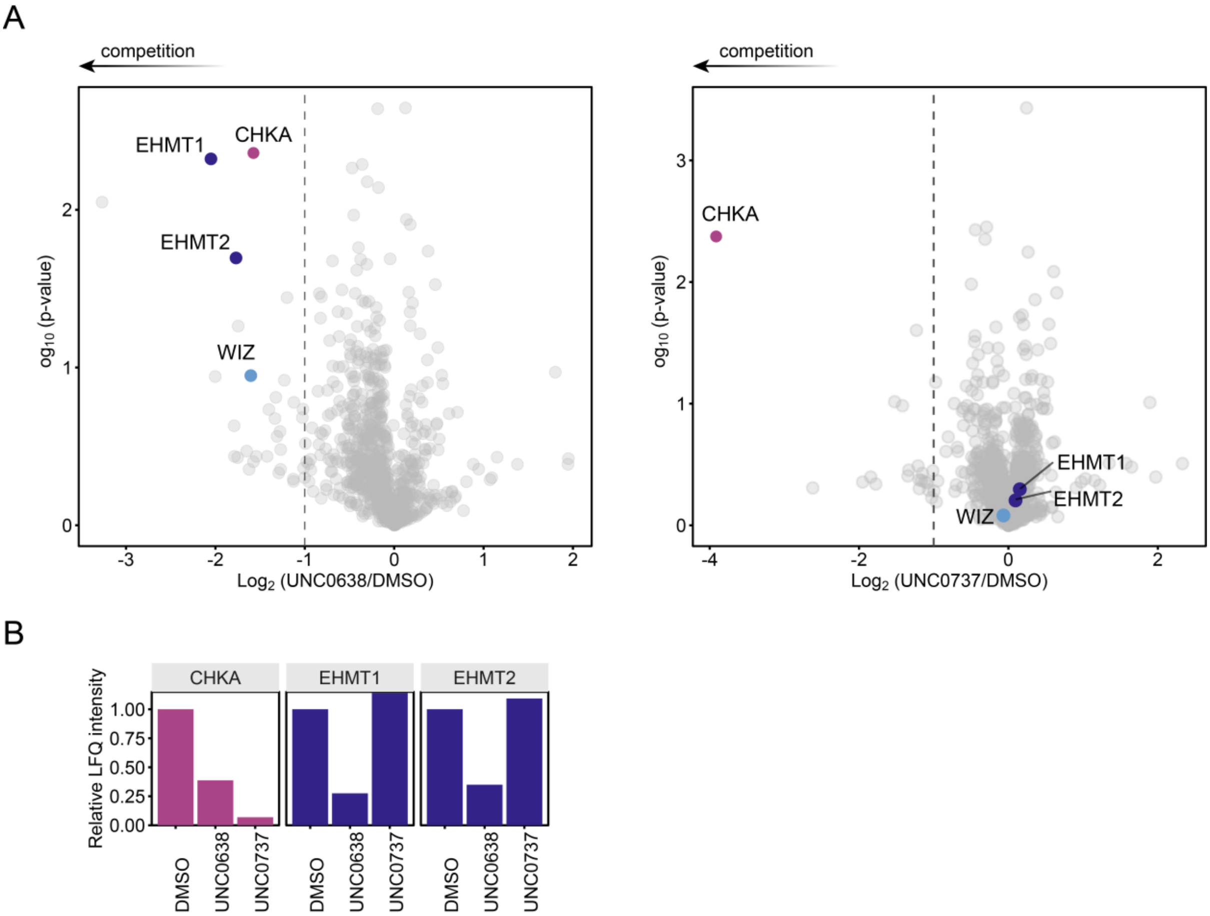


**Figure S8**: Affinity-based chemoproteomics using UNC0965 functionalised affinity matrix and 20 µM UNC0638 or UNC0737 as competitors in PBMC cell extract. A) Volcano plots show CHKA as shared target of UNC0638 and UNC0737. Dashed lines represent $\log_2$ competition < -1 (p-values from two-sided two sample t-test, n = 2). 922 proteins were identified. B) CHKA mean LFQ intensity is reduced stronger after competition of 20 µM UNC0737 compared to the same concentration of UNC0638. EHMT1 (GLP) and EHMT2 (G9a) only exhibit reduced binding to the matrix in presence of UNC0638.

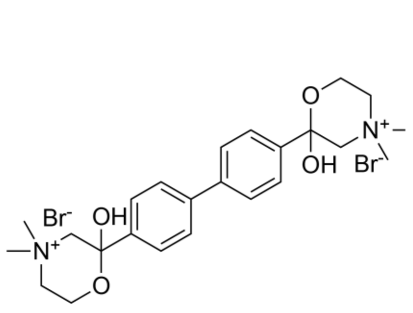

HC3

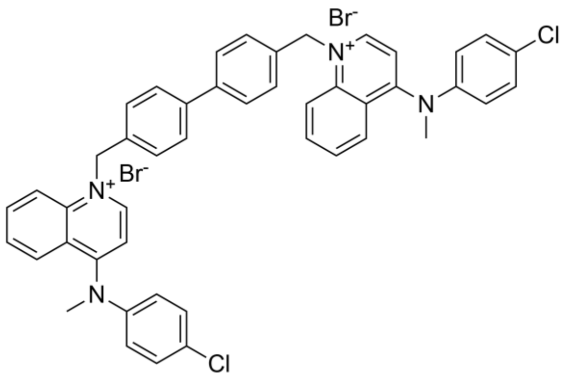

RSM-932A (TCD-717)

**Figure S9:** Chemical structures of HC3 and RSM-932A (TCD-717).

## References:


[1] K. D. Konze, S. G. Pattenden, F. Liu, D. Barsyte-Lovejoy, F. Li, J. M. Simon, I. J. Davis, M. Vedadi, J. Jin, *ChemMedChem* **2014**, *9*, 549-553.

[2] S. Scheer, S. Ackloo, T. S. Medina, M. Schapira, F. Li, J. A. Ward, A. M. Lewis, J. P. Northrop, P. L. Richardson, H. Ü. Kaniskan, Y. Shen, J. Liu, D. Smil, D. McLeod, C. A. Zepeda-Velazquez, M. Luo, J. Jin, D. Barsyte-Lovejoy, K. V. M. Huber, D. D. De Carvalho, M. Vedadi, C. Zaph, P. J. Brown, C. H. Arrowsmith, *Nat. Commun.* **2019**, *10*, 19.

[3] J. Cox, M. Mann, *Nat. Biotechnol.* **2008**, *26*, 1367-1372.

[4] S. Tyanova, T. Temu, P. Sinitcyn, A. Carlson, M. Y. Hein, T. Geiger, M. Mann, J. Cox, *Nat. Methods* **2016**, *13*, 731-740.

[5] E. Gasteiger, C. Hoogland, A. Gattiker, S. e. Duvaud, M. R. Wilkins, R. D. Appel, A. Bairoch, *Protein identification and analysis tools on the ExPASy server*, Springer, **2005**.

[6] C. Vonrhein, C. Flensburg, P. Keller, A. Sharff, O. Smart, W. Paciorek, T. Womack, G. Bricogne, *Acta Cryst. D* **2011**, *67*, 293-302.

[7] P. Evans, *Acta Cryst. D* **2011**, *67*, 282-292.

[8] A. J. McCoy, R. W. Grosse-Kunstleve, P. D. Adams, M. D. Winn, L. C. Storoni, R. J. Read, *J. Appl. Crystallogr.* **2007**, *40*, 658-674.

[9] G. N. Murshudov, P. Skubák, A. A. Lebedev, N. S. Pannu, R. A. Steiner, R. A. Nicholls, M. D. Winn, F. Long, A. A. Vagin, *Acta Crystallogr. Sect. D. Biol. Crystallogr.* **2011**, *67*, 355-367.

[10] P. Emsley, B. Lohkamp, W. G. Scott, K. Cowtan, *Acta Crystallogr. Sect. D. Biol. Crystallogr.* **2010**, *66*, 486-501.

[11] A. L. Chernobrovkin, C. Cázares-Körner, T. Friman, I. M. Caballero, D. Amadio, D. Martinez Molina, *SLAS Discov.* **2021**, *26*, 534-546.

[12] J. A. Hendricks, N. Beaton, A. Chernobrovkin, E. Miele, G. M. Hamza, P. Ricchiuto, R. C. Tomlinson, T. Friman, C. Borenstain, B. Barlaam, S. Hande, M. L. Lamb, C. De Savi, R. Davies, M. Main, J. Hellner, K. Beeler, Y. Feng, R. Bruderer, L. Reiter, D. M. Molina, M. P. Castaldi, *ACS Chem. Biol.* **2022**, *17*, 54-67.

[13] S. Kostidis, R. D. Addie, H. Morreau, O. A. Mayboroda, M. Giera, *Anal. Chim. Acta* **2017**, *980*, 1-24.

[14] Y. Sundström, M.-M. Shang, S. K. Panda, C. Grönwall, F. Wermeling, I. Gunnarsson, I. E. Lundberg, M. Sundström, P.-J. Jakobsson, L. Berg, *Transl. Res.* **2021**, *229*, 69-82.

[15] M.-A. Bray, S. Singh, H. Han, C. T. Davis, B. Borgeson, C. Hartland, M. Kost-Alimova, S. M. Gustafsdottir, C. C. Gibson, A. E. Carpenter, *Nat. Protoc* **2016**, *11*, 1757-1774.

[16] M. H. Woehrmann, W. M. Bray, J. K. Durbin, S. C. Nisam, A. K. Michael, E. Glassey, J. M. Stuart, R. S. Lokey, *Mol. Biosyst.* **2013**, *9*, 2604-2617.

[17] A. Pahl, B. Schölermann, P. Lampe, M. Rusch, M. Dow, C. Hedberg, A. Nelson, S. Sievers, H. Waldmann, S. Ziegler, *Cell Chem. Biol.* **2023**, *30*, 839-853.e837.
